# Assembly and comparative analysis of the mitochondrial genome of *Ceratophyllum demersum* L.

**DOI:** 10.3389/fpls.2025.1704888

**Published:** 2025-10-27

**Authors:** Hang Yin, Xiaogang Dai

**Affiliations:** ^1^ State Key Laboratory for Tree Genetics and Breeding, Co-Innovation Center for Sustainable Forestry in Southern China, Key Laboratory of Tree Genetics and Biotechnology of Educational Department of China, Key Laboratory of Tree Genetics and Breeding of Jiangsu Province, Nanjing Forestry University, Nanjing, China; ^2^ College of Ecology and Environment, Nanjing Forestry University, Nanjing, China

**Keywords:** *Ceratophyllum demersum*, mitochondrial genome, RNA editing, repeat elements, phylogenetic analysis

## Abstract

Mitochondria are the powerhouse of eukaryotic cells, whose genomes feature unique structural characteristics and evolutionary significance. *Ceratophyllum demersum* is a widely distributed aquatic plant that holds special position in the aquatic ecosystem. In this study, we assembled the mitochondrial genome (mitogenome) of *C. demersum* from the PacBio HiFi sequencing data, yielding three complete circular chromosomes of lengths 285,151 bp, 208,195 bp and 101,944 bp. The three molecules contain 65 unique genes comprising 40 protein coding genes (PCGs), 3 rRNA genes, and 22 tRNA genes. The frequent recombination of mitogenome is driven by the non-tandem repetitive sequences. Genome comparison showed that the content of non-tandem repeats in the mitogenome of the algae-like *C. demersum* was significantly higher than that in terrestrial angiosperms. In monocotyledonous and dicotyledonous plants, there is a significant loss of large ribosomal and small subunit genes. By contrast, *C. demersum* possesses all 24 core PCGs and inherits a similar number of PCGs as the ancient angiosperms of Magnoliaceae and Chloranthaceae, with only three variable PCGs (*rpl6*, *rps8*, and *rps19*) lost during evolution, suggesting a special evolutionary position of *C. demersum* in angiosperms. Phylogenetic analyses support the monophyly of Ceratophyllales and Chloranthales and places this clade as sister to a combined monocot–eudicot group. These findings offer new insights and propose alternative hypotheses for reconstructing the early evolutionary history of angiosperms.

## Introduction

As the most diverse group within the plant kingdom, angiosperms (also known as flowering plants) are not only a crucial component of Earth’s biosphere but also play an irreplaceable role in maintaining global ecological balance (Yang et al., 2020b). The early evolutionary phase of angiosperms exhibited rapid radiation and diversification (Steemans et al., 2009; Li et al., 2019). However, due to the scarcity of reliable fossil evidence, the phylogenetic relationships among different divergent lineages during this period have yet to yield clear and unified conclusions (Friedman, 2009; Guo et al., 2021). With the advent of omics, researchers have extensively utilized plastid genomes (plastomes) and single-copy nuclear genes for studies on early angiosperm evolution (Zeng et al., 2014; Yang et al., 2020a; Guo et al., 2022; Hu et al., 2023), yet only a few have employed mitochondrial genomes (mitogenomes) for large-scale phylogenetic analyses (Xue et al., 2021; Hu et al., 2023; Lin et al., 2025).

As an organelle with independent genetic material in both plant and animal cells, mitochondria have undergone an exceptionally complex evolutionary trajectory within eukaryotes since their endosymbiotic origin (Gray et al., 1999; Sun et al., 2024). Compared to the compact and structurally conserved animal mitogenomes, plant mitogenomes exhibit a unique “evolutionary paradox”: extremely low sequence mutation rates (far below those of animal mitochondria) coupled with frequent genomic recombination (Christensen, 2021). These recombination events maintain mitochondrial DNA (mtDNA) stability when damaged but also continuously drive mitogenome evolution (Wu et al., 2020), resulting in enormous size variation among plant mitogenomes (66 kb–18.99 Mb) (Skippington et al., 2015; Huang et al., 2025). Very recently, *Cathaya argyrophylla* was reported to possess the super-large record breaking mitogenome, with size up to 18.99 Mb (Huang et al., 2025). Even closely related species exhibit substantial mitogenome size variation, such as the 45-fold difference between *Silene latifolia* (253 kb) and *S. conica* (11.3 Mb) in the Caryophyllaceae (Wu et al., 2020), and the 7-fold difference between *Cucumis melo* (2.9 Mb) and *Citrullus lanatus* (379 kb) in the Cucurbitaceae (Alverson et al., 2010). Furthermore, frequent recombination mediated by repetitive sequences leads to highly diverse mitogenome structures (Gualberto and Newton, 2017), including multi-circular, single-circular, linear, branched linear, branched circular, and complex structures combining multiple forms (Zou et al., 2022; Yang et al., 2023). Moreover, distinct structural types exhibit markedly different distribution frequencies within cells (Kozik et al., 2019). The complex structure of plant mitochondria poses significant challenges for complete genome assembly (Liu et al., 2024; Wang et al., 2025). Additionally, difficulties in effectively isolating mtDNA from total cellular DNA, low sequence conservation among different species, and extensive homologous sequences shared with nuclear and plastid genomes further complicate mitogenome assembly.

With the advancement of sequencing technology, the popularity of third-generation long-read sequencing data for plant whole genomes has surged dramatically (Bi et al., 2025). The extended read lengths now make complete mitogenome assembly feasible. Recently, several assembly tools targeting plant mitogenomes have been developed, including PMAT (Bi et al., 2024a; Han et al., 2025), TIPPo (Xian et al., 2025), OATK (Zhou et al., 2025), and HiMT (Tang et al., 2025). Among these, PMAT does not require pre-assembly filtering of mitochondrial reads and directly utilizes copy number differences among chloroplast, mitochondrial, and nuclear genomes within whole-genome assembly to obtain a complete mitochondrial assembly. In contrast, the other tools need to first enrich mitochondrial reads using kmer-based methods before assembly, which significantly improves run speed but renders the method susceptible to interference from non-coding mitochondrial DNA (NUMT) and mitochondrial transfer proteins (MTPT) sequences, potentially leading to misclassification and structural loss during assembly. Early plant phylogenetic analyses predominantly relied on plastid genomes whereas the maternally inherited mitogenome was underutilized. The emergence of these assembly tools now enables phylogenetic analyses utilizing extensive plant mitogenome data. Angiosperm mitogenomes contain 43 relatively conserved protein-coding genes that provide rich phylogenetic information (Mower, 2020; Bi et al., 2024b), offering evidence for unresolved issues in plastid and nuclear genes (Xue et al., 2021; Lin et al., 2025). Mitochondrial genes perform poorly in phylogenetic analyses conducted at the family or genus level given the slow nucleotide substitution rates compared to those of plastid and nuclear genomes (Knoop, 2004; Yurina and Odintsova, 2016); however, this feature is advantageous for reconstructing the phylogenetic relationships of ancient lineages (Lin et al., 2025).

Mesangiospermae are generally recognized to comprise five major lineages, namely Chloranthales, Magnoliids, Monocots, Ceratophyllales, and Eudicots, but the evolutionary relationships among these lineages remain unresolved (Yang et al., 2020a; Guo et al., 2022). Ceratophyllaceae is a core family within the Ceratophyllales order of angiosperms, comprising 1 genus and 7 species widely distributed in freshwater environments globally (Yang et al., 2020b). The unique morphological and molecular biological characteristics of hornworts (*Ceratophyllum demersum*), such as their rootless growth and distinctive floral development, have long made their phylogenetic position controversial (Li et al., 2019; Guo et al., 2022; Hu et al., 2023). Analyzing Ceratophyllid’s genetic information is crucial for understanding the family’s evolution, phylogeny, and sustainable utilization. While chloroplast and nuclear genomes of *Ceratophyllum* have been published (Yang et al., 2020b; Ru et al., 2025), the mitogenome remains unreleased. This study employed PacBio HiFi sequencing technology to assemble the *C. demersum* mitogenome into three single-stranded circular structures, providing the first mitochondrial reference genome for plants in the Ceratophyllaceae family. The sequence annotation, comparative genomics, and phylogenetic analyses of this mitogenome offer crucial insights for accurately reconstructing the early evolutionary history of angiosperms.

## Materials and methods

### Plant material, DNA extraction and sequencing

Fresh leaves of *C. demersum* were collected from the Xuanwu Lake (Nanjing, China; geographic coordinates: 32°05’01” N, 118°47’34” E) and immediately frozen in liquid nitrogen for subsequent DNA extraction. Genomic DNA isolation was performed with the Hi-DNAsecure Plant Kit (Tiangen Biotech, Beijing, China). The DNA quality was assessed by agarose gel electrophoresis and a Nanodrop 2000 ultraviolet spectrophotometer (ThermoFisher, Massachusetts, USA). Following quality verification, high-integrity genomic DNA was utilized to prepare 15-kb sequencing libraries using the SMRTbell Express Template Prep Kit 2.0 (PacBio Biosciences, California, USA). Ultimately, high-fidelity (HiFi) sequencing data were generated on the PacBio Revio sequencing platform (Pacific Biosciences, California, USA).

### Mitogenome assembly and annotation

The PacBio HiFi sequencing data from *C. demersum* were input into PMAT2 for mitogenome assembly (Han et al., 2025), with parameters configured as ‘autoMito -t hifi -m -T 50’. The raw assembly graph generated from PMAT was subsequently visualized and disentangled using Bandage (Wick et al., 2015), which yielded three distinct single-circular chromosomes for downstream annotation. The initial annotation of the *C. demersum* mitogenome was conducted using the online platform PMGA (http://www.1kmpg.cn/pmga/) (Li et al., 2025a). Then, tRNAscan-SE v2.0 (Chan et al., 2021) and BLASTn (Camacho et al., 2009) were used to check all annotated tRNA and rRNA genes, respectively. All protein-coding genes (PCGs), tRNAs, and rRNAs were subjected to manual inspection and correction using MacVector v18.8 to ensure annotation accuracy. Finally, the mitogenomic map of *C. demersum* was drawn using the online tool PMGmap (http://www.1kmpg.cn/pmgmap) (Zhang et al., 2024).

### Identification of repeat elements

Simple sequence repeats (SSRs) within the *C. demersum* mitogenome were detected using SSRMMD (Gou et al., 2020) with the following parameter settings: ‘-mo 1 = 10,2 = 5,3 = 4,4 = 3,5 = 3,6 = 3 -ss 1 -e 1’. Tandem repeats were identified using Tandem Repeats Finder v4.09 (Benson, 1999) with the basic mode. The parameters for alignment were 2, 7, 7 for match (matching weight), mismatch (mismatching penalty), and delta (indel penalty), respectively, and the minimum alignment score was set at 50. The minimum and maximum period size were set at 10 and 2000, respectively. Furthermore, we utilized the Python script ROUSFinder2.0.py (https://github.com/flydoc2000/ROUSfinder) (Wynn and Christensen, 2019) to detect non-tandem dispersed repeats in the *C. demersum* mitogenome, with the minimum identity, E-value and minimum repeat size configured to 98%, 10, and 50 bp, respectively. The mitogenomic distribution of these non-tandem repeats were visualized using Circos (Krzywinski et al., 2009).

### Whole-mitogenome collinearity analysis

To investigate the evolutionary characteristics of the *C. demersum* mitogenome, we downloaded the mitogenomes of seven other angiosperm species from the NCBI Nucleotide Database, including *Hedyosmum orientale*, *Nymphaea colorata*, *Cinnamomum chekiangense*, *Liriodendron tulipifera*, *Butomus umbellatus*, *Stephania japonica*, and *Nelumbo nucifera*. The NCBI accession numbers for these mitogenomes are provided in **Supplementary Table S1**. The nucmer program integrated in MUMmer v3.23 (Kurtz et al., 2004) was used to align the seven mitogenomes against the *C. demersum* mitogenome. Subsequently, the delta-filter program was employed to filter the alignment results generated by nucmer, with thresholds set as a minimum identity of > 80% and an alignment length of > 100 bp (Bi et al., 2022). The show-coords program was utilized to parse the delta alignment outputs and display summarized information for each alignment, including position and percent identity. A custom R script was ultimately used to visualize collinearity between the mitogenomes of *C. demersum* and the other seven plants.

### Detection of RNA editing sites

RNA editing sites within the *C*. *demersum* mitogenome were detected using Deepred-Mt (Edera et al., 2021), a neural network-based tool specifically designed for predicting C-to-U editing sites in angiosperm mitogenomes. To improve prediction accuracy, we extracted the coding sequences of each PCG, together with their 20 bp of upstream and downstream flanking regions, and used these sequences as input for Deepred-Mt. A probability threshold of 0.9 was applied to ensure high-confidence predictions (Han et al., 2024).

### Phylogenetic analysis

To resolve the phylogenetic position of *C. demersum* among angiosperms using mitogenomes, the coding sequences (CDS) of 32 plant mitogenomes were retrieved from the NCBI Nucleotide Database (**Supplementary Table S1**), including Bryophytes, Gymnosperms, the ANA grade, and five major clades of Mesangiospermae (Chloranthales, Ceratophyllales, Magnoliids, Monocots, and Eudicots). A total of 26 conserved PCGs were extracted from these mitogenomes using custom scripts. Each gene dataset was individually aligned with MAFFT v7.525 (Katoh and Standley, 2013) under default parameters, followed by trimming of poorly aligned regions using Gblocks 0.91b (Talavera and Castresana, 2007) with the least stringent settings to reduce noise. The trimmed alignment files for each gene were then concatenated in order using custom scripts. For phylogenetic inference, ModelFinder (integrated in IQ-TREE v2.4.0) (Minh et al., 2020) was employed to select the best-fit partition model (GTR+F+I+G4), and a maximum likelihood (ML) phylogenetic tree was constructed using RAxML v8.2.13 (Stamatakis, 2014). The analysis initiated with 1000 random starting trees to search for the tree with the highest likelihood, and non-parametric bootstrap support values were calculated based on 1000 iterations. Finally, the resulting phylogenetic tree was visualized using the online platform iTOL (https://itol.embl.de/) (Letunic and Bork, 2019).

## Results

### Assembly and general characteristics of the *C*. *demersum* mitogenome

**Figure 1:**
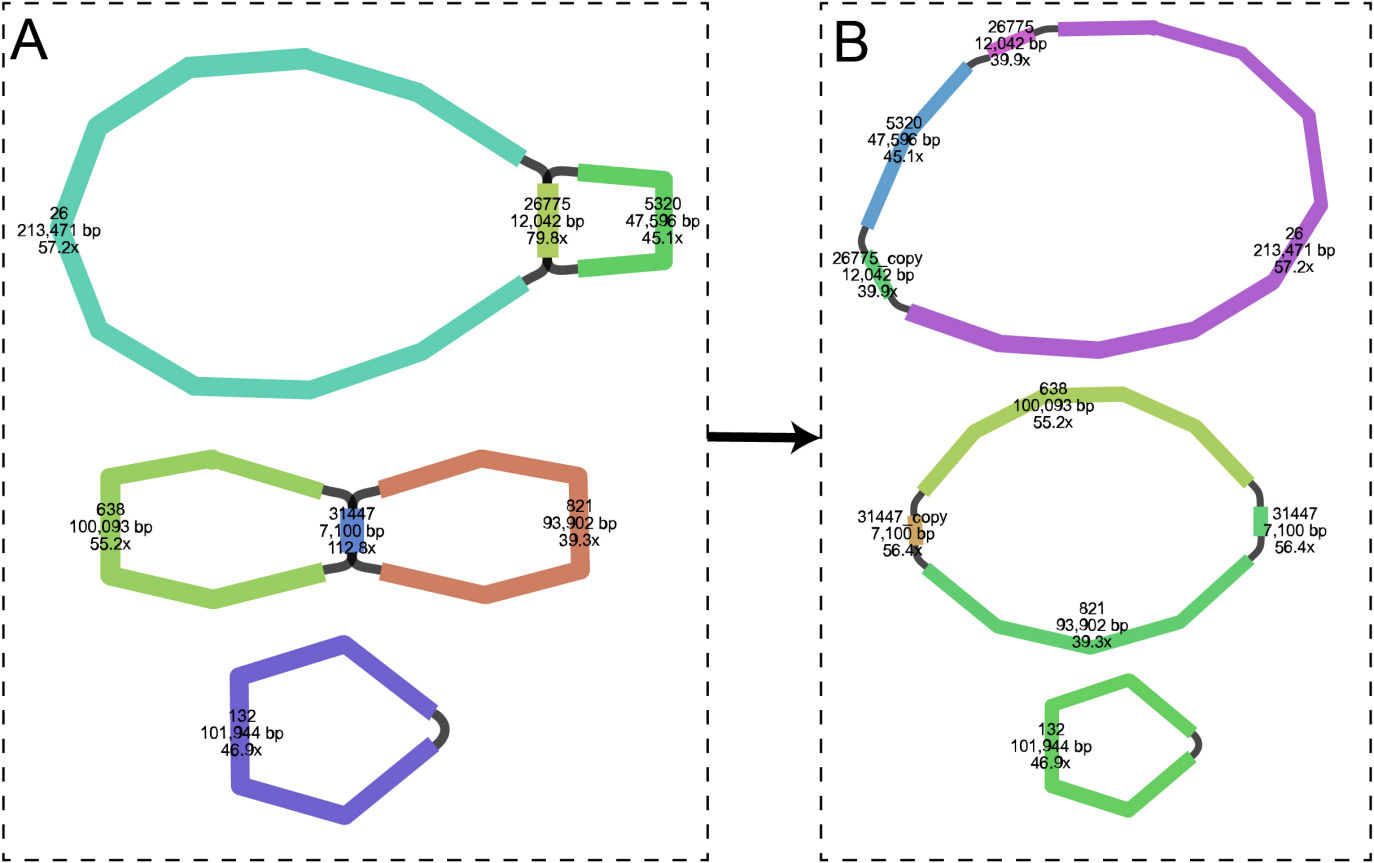
Assembly graph of the *C*. *demersum* mitogenome. **(A)** Raw assembly graph generated by PMAT2. **(B)** Master assembly graph disentangled by Bandage software.

To assemble the complete mitogenome of *C*. *demersum*, a total of 4.61 Gb PacBio HiFi sequencing data were generated by PacBio Revio platform. The maximum and average lengths of the HiFi reads are 54,894 bp and 21,894 bp, respectively. Leveraging highly accurate long-read sequencing data, the *C*. *demersum* mitogenome was successfully assembled into three circular chromosomes (**Figure 1**), with a total length of 595,290 bp and an overall GC content of 49.64% (**Table 1**). Among the three circular chromosomes, the largest one (mtChr1) was 285,151 bp in length, followed by mtChr2 with 208,195 bp, and the smallest (mtChr3) was only 101,944 bp.

**Table 1:** General characteristics of the *C. demersum* mitogenome.

Statistical information	Total mitogenome	mtChr1	mtChr2	mtChr3
Accession number	OR366023-25	OR366023	OR366024	OR366025
Length (bp)	595,290	285,151	208,195	101,944
GC content	49.64%	49.55%	49.74%	49.70%
Number of total genes	77	40	23	14
Number of PCGs	40	14	15	11
Number of rRNA genes	3	1	2	0
Number of tRNA genes	34	25	6	3
Total length of genes (bp)	43,120 (7.24%)	16,926 (5.87%)	16,418 (7.89%)	9,776 (9.59%)
Total length of PCGs (bp)	34,608 (5.81%)	11,214 (3.93%)	13,836 (6.65%)	9,558 (9.38%)
Total length of tRNAs (bp)	2,597 (0.44%)	1,932 (0.68%)	447 (0.21%)	218 (0.21%)
Total length of rRNAs (bp)	5,915 (0.99%)	3,780 (1.33%)	2,135 (1.03%)	0
Number of cis-spliced introns	21	5	11	5

**Figure 2:**
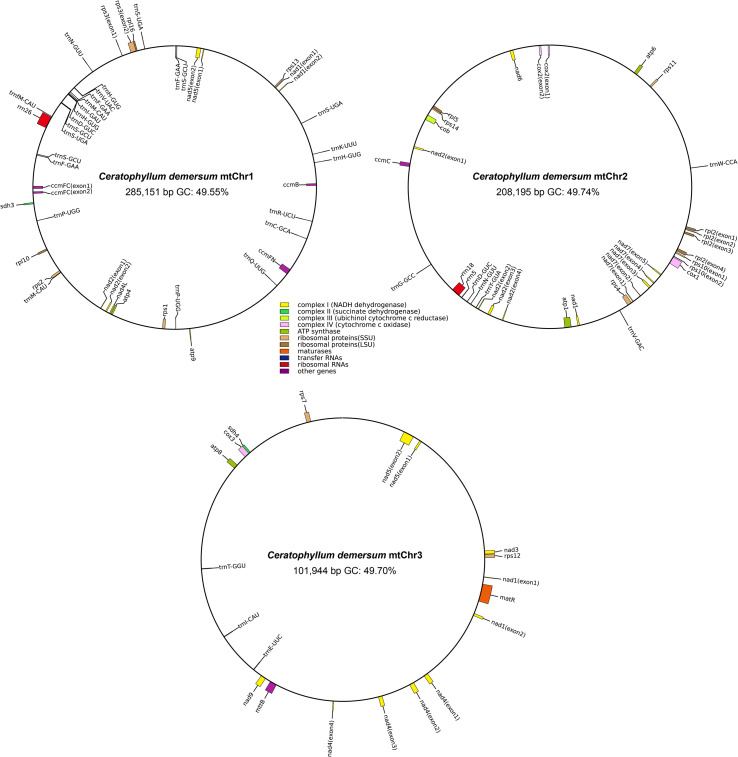
The multi-circular genome map of the *C*. *demersum* mitogenome. Genes are depicted with different colors based on their specific functions.

The *C*. *demersum* mitogenome encodes a total of 65 unique genes (**Figure 2**; **Supplementary Table S2**), comprising 40 PCGs spanning 43,120 bp, 3 rRNA genes (5,915 bp), and 22 tRNA genes (2,597 bp). Among these, 8 tRNA genes exist as multiple copies, specifically *trnD*-GUC, *trnF*-GAA, *trnH*-GUG, *trnM*-CAU, *trnN*-GUU, *trnP*-UGG, *trnS*-GCU, and *trnS*-UGA. A total of 26 introns were identified across 10 PCGs, namely *ccmFC*, *cox2*, *nad1*, *nad2*, *nad4*, *nad5*, *nad7*, *rpl2*, *rps3*, and *rps10*. Of these introns, five are *trans*-spliced, distributed among the *nad1*, *nad2*, and *nad5* genes, while the remaining are *cis*-spliced. Notably, most intron-containing genes are localized to a single chromosome, whereas *nad1*, *nad2*, and *nad5* are dispersed across different chromosomes. Furthermore, among the 40 PCGs, the majority use the standard ATG as the start codon. Exceptions include five PCGs (*cox1*, *nad1*, *nad4L*, *rps10*, and *sdh4*) which use ACG as the start codon, a modification resulting from RNA editing events (**Supplementary Table S2**). Notably, the start codons for *mttB* and *rpl16* remain undetermined. Most genes use TAA (15 genes), TGA (13 genes), or TAG (9 genes) as the stop codon. In contrast, *atp9* terminates with CGA, whereas *atp6* and *rps11* use CAA as the stop codon, and both cases are also attributed to RNA editing events.

### Analysis of repeat sequences

**Figure 3:**
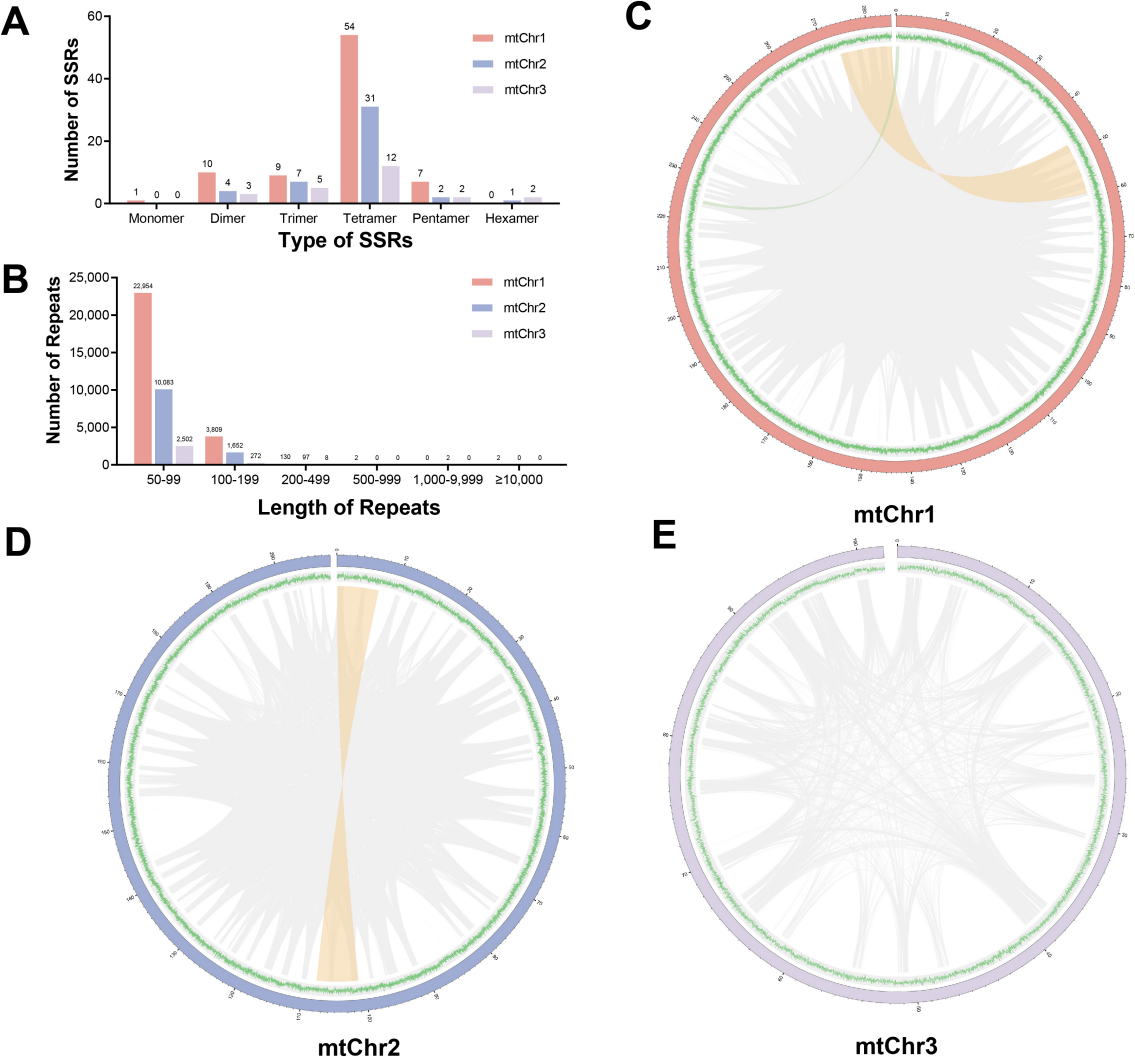
Repeat elements identified in the *C*. *demersum* mitogenome. **(A)** The frequency of simple sequence repeats (SSRs). **(B)** The frequency of non-tandem dispersed repeats. Distribution of non-tandem dispersed repeats in **(C)** mtChr1, **(D)** mtChr2, and **(E)** mtChr3. Repeats with lengths exceeding 1000 bp are shown in orange, whereas those with lengths range from 500 to 1000 bp are shown in green.

Repeat sequences in plant mitogenomes are pivotal for driving genome structural evolution, mediating genetic recombination events, and serving as valuable molecular markers in phylogenetic and population genetic studies. In this study, a total of 150 SSRs were detected, with 81 in mtChr1, 45 in mtChr2, and 24 in mtChr3, respectively. Regarding the classification of SSR repeat units, these SSRs include 1 mono-, 17 di-, 21 tri-, 97 tetra-, 11 penta-, and 3 hexameric repeats (**Figure 3A**), with the tetrameric repeat of AAGC/CTTG found to be more abundant than others. Additionally, we identified a total of 1,034 tandem repeats in the *C. demersum* mitogenome, with 481 repeats located on mtChr1, 368 on mtChr2, and 185 on mtChr3 (**Supplementary Table S3**). The largest tandem repeat is 354 bp in length, with the majority (84.72%) being shorter than 100 bp and 158 tandem repeats exceeding 100 bp. Most tandem repeats (86.07%) have four or fewer copies, while 144 repeats (13.93%) contain more than four copies. Notably, the highest copy number was observed in a 58-bp tandem repeat, which was tandemly repeated 16 times.

Remarkably, we identified a total of 41,513 pairs of non-tandem dispersed repeats (5,558 repeat units) with lengths ≥50 bp in the *C*. *demersum* mitogenome (**Figure 3B**). Specifically, mtChr1 harbors 26,897 repeats and 3,152 repeat units (**Figure 3C**; **Supplementary Table S4**), with a cumulative length of 96,717 bp (33.92% of mtChr1); mtChr2 contains 11,834 repeats and 1,748 repeat units (**Figure 3D**), totaling 62,759 bp (30.14% of mtChr2); and mtChr3 has 2,282 repeats and 658 repeat units (**Figure 3E**), with a combined length of 22,782 bp (22.35% of mtChr3). Collectively, these dispersed repeats span 182,258 bp, representing 30.62% of the entire *C. demersum* mitogenome. Among these dispersed repeats, approximately 85.6% (35,539 repeats) are shorter than 100 bp (**Figure 3B**), whereas only 6 repeats exceed 500 bp in length. Specifically, four of these large repeats (≥500 bp) are located on mtChr1, with the longest reaching 12,042 bp; two are found on mtChr2, with the maximum length of 7,102 bp; and no repeats longer than 500 bp are detected on mtChr3. Additionally, mtChr1 has an average repeat size of 89.56 bp, mtChr2 of 89.77 bp, mtChr3 of 75.98 bp, and the average repeat size of the whole mitogenome is 85.11 bp. The average copy number of repeats is 8.53 in mtChr1, 6.77 in mtChr2, 4.23 in mtChr3, and 6.51 for the entire mitogenome.

### Analysis of mitogenome collinearity

**Figure 4:**
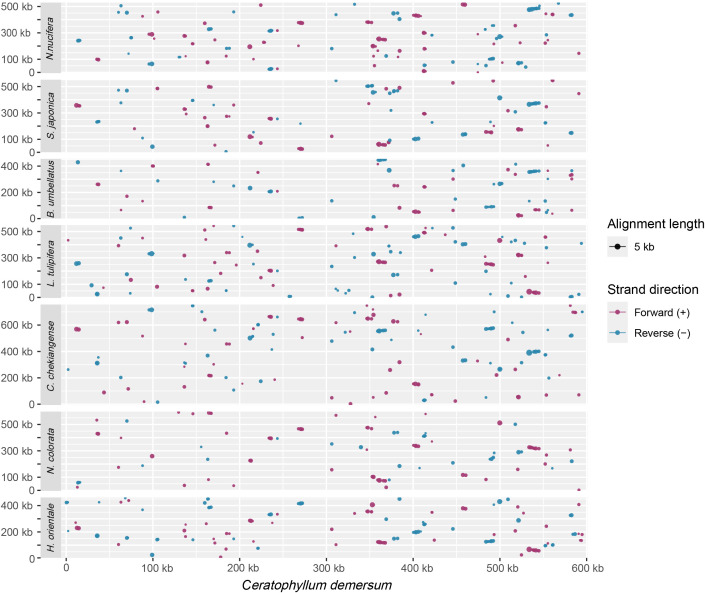
Whole mitogenome collinearity among eight angiosperm species. The *C. demersum* mitogenome was set as reference. Red and blue lines represent forward and reverse syntenic regions, respectively.

Frequent rearrangement is the pivotal driving force behind the evolution of plant mitogenomes. In this study, we conducted a comprehensive comparative analysis of the *C*. *demersum* mitogenome with seven other angiosperm mitogenomes. As shown in **Supplementary Table S5**, when compared with *H. orientale*, a total of 129 local colinear blocks (LCBs) were identified, accounting for 20.46% (96,525 bp) of its mitogenome; for *N. colorata*, 94 LCBs were found, covering 11.63% (71,793 bp) of its mitogenome; in the case of *C. chekiangense*, 143 LCBs were detected, making up 15.14% (113,609 bp) of its mitogenome; for *B. umbellatus*, 92 LCBs account for 13.68% (61,664 bp) of its mitogenome; when comparing with *S. japonica*, 117 LCBs cover 17.25% (95,730 bp) of its mitogenome; and for *N. nucifera*, 152 LCBs make up 21.19% (111,194 bp) of its mitogenome. The overall collinearity among the eight analyzed mitogenomes is remarkably low (**Figure 4**). The collinear regions between *N. nucifera*, *L. tulipifera*, *H. orientale*, and *C. demersum* account for only approximately 20% of their respective entire mitogenomes, while the proportions of collinear regions in *N. colorata* and *B. umbellatus* are even less than 15%. These findings suggest that the mitogenomes of major core clades of angiosperms have endured extensive genomic rearrangements during evolution, leading to a pronounced loss of collinearity among mitogenomes.

### Analysis of RNA editing events

**Figure 5:**
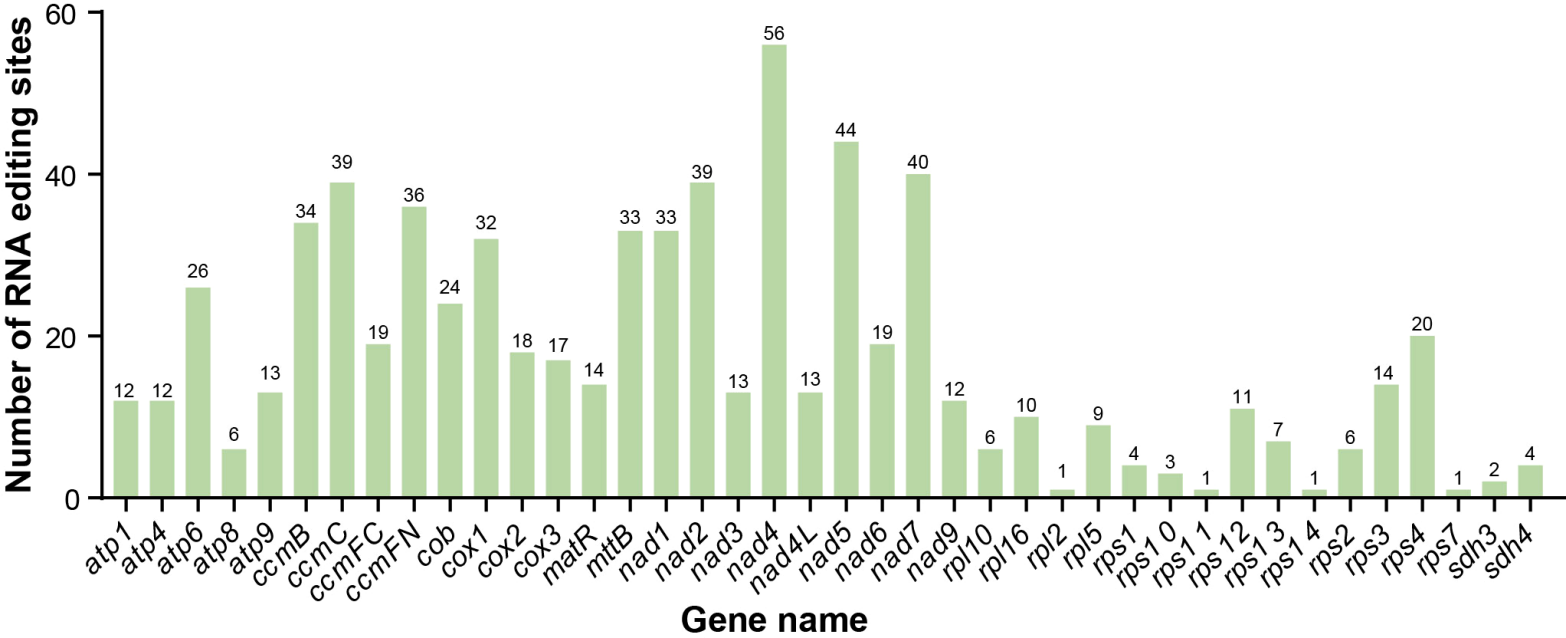
Prediction of RNA editing events in all PCGs of the *C. demersum* mitogenome.

RNA editing in plant mitogenomes is critical for accurate gene expression and mitochondrial functional integrity, as it post-transcriptionally corrects genomic “errors” and modulates RNA function, which is essential for core processes like oxidative phosphorylation. In this study, a total of 701 C-to-U RNA editing sites were identified across 40 PCGs in the *C*. *demersum* mitogenome (**Supplementary Table S6**). The majority of these editing events occurred at the second codon position (448 sites), followed by the first position (212 sites), with only 41 sites located at the third codon position. As shown in **Figure 5**, *nad4* (56 sites) exhibits the highest number of RNA editing sites, followed by *nad5* and *nad7*, both of which contain over 40 editing sites. In contrast, 13 genes (*atp8*, *rpl2*, *rpl5*, *rpl10*, *rps1*, *rps2*, *rps7*, *rps10*, *rps11*, *rps13*, *rps14*, *sdh3*, and *sdh4*) each possess fewer than 10 editing sites. Among these, *rpl2*, *rps7*, and *rps14* contain only one RNA editing site each.

### Phylogenetic analysis

**Figure 6:**
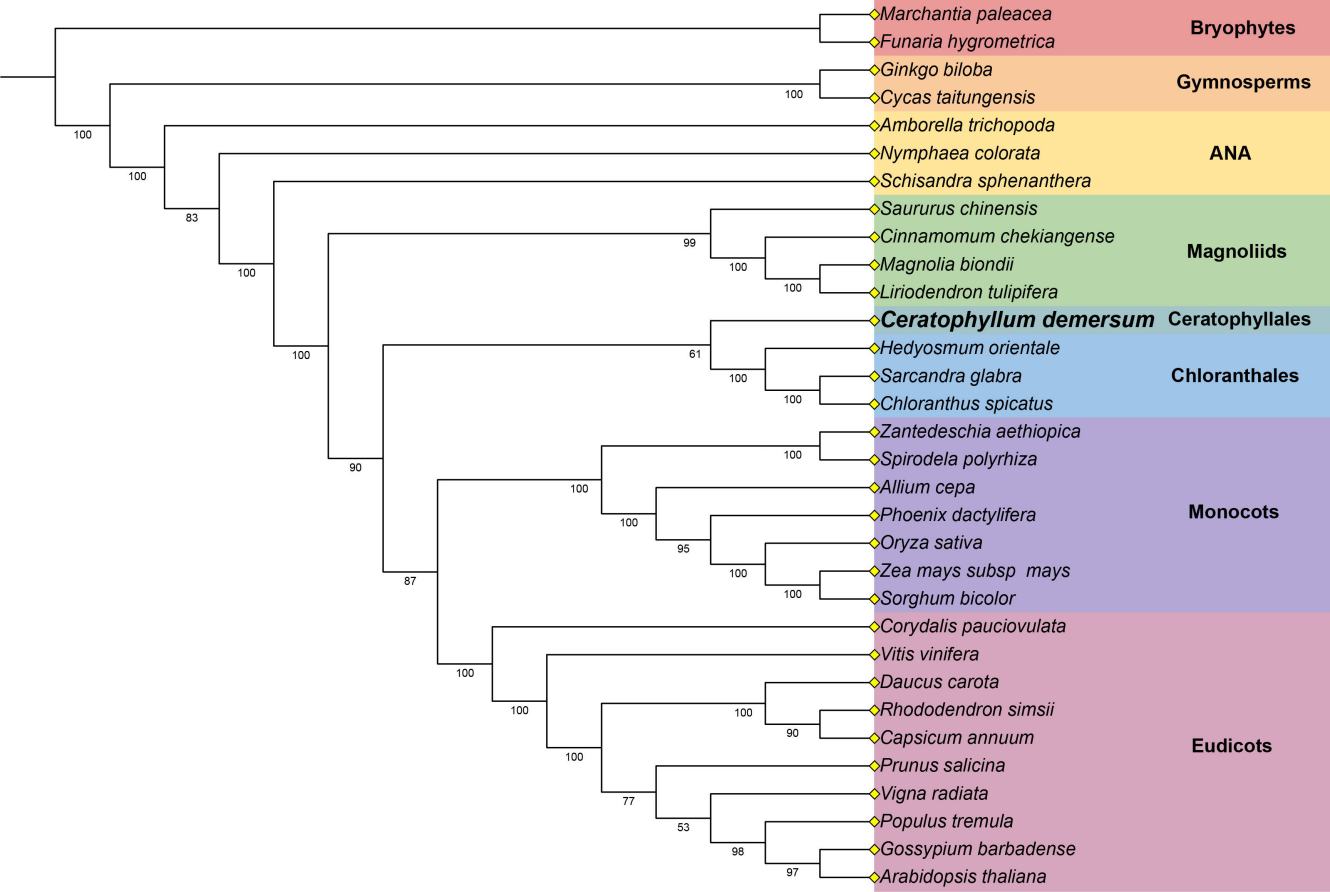
The ML tree of 32 plant species based on 25 conserved mitochondrial PCGs. *Marchantia paleace*a and *Funaria hygrometrica* were select as the outgroup. The bootstrap values are displayed on each branch, and the colors indicate the taxonomic groups of each species.

The slow evolutionary rate of plant mitochondrial genes facilitates in-depth insights into the phylogenetic relationships of early angiosperms. In this study, we conducted phylogenetic analyses using 25 conserved mitochondrial PCGs (*atp1*, *atp4*, *atp6*, *atp8*, *atp9*, *ccmB*, *ccmC*, *ccmFc*, *ccmFn*, *cob*, *cox1*, *cox2*, *cox3*, *matR*, *nad1*, *nad2*, *nad3*, *nad4*, *nad4L*, *nad5*, *nad6*, *nad7*, *nad9*, *rps3*, and *rps12*) from 32 species, with two bryophyte species designated as the outgroup. As shown in **Figure 6**, the ML tree strongly supports the ANA grade as the basal clade of angiosperms, which is resolved as the sister group to all other angiosperms. The ML tree emphasizes the magnoliids as the basal clade among the five Mesangiospermae clades of angiosperms. It also provides strong support for a close phylogenetic relationship between *C*. *demersum* and the other three species within Chloranthales. Notably, this clade (comprising Chloranthales and Ceratophyllales) is resolved as the sister group to the clade encompassing monocots and eudicots. Most nodes in the ML tree exhibit bootstrap values exceeding 80%, indicating high reliability of the phylogenetic topology.

## Discussion

### Variations in size and structure of plant mitogenomes

Plant mitogenomes have long exhibited numerous unique characteristics not found in animal mitogenomes. Animal mitogenomes typically consist of circular DNA molecules ranging from 15 to 17 kb in length, whereas most plant mitogenomes are dynamically composed of varying proportions of circular, linear, or branched DNA molecules, with considerable length variation across different plant groups (Kozik et al., 2019; Wu et al., 2020; Li et al., 2025b). In this study, the mitogenome of *C*. *demersum* exhibits a multi-ring structure composed of three independently replicating circular chromosomal molecules, with a total length of approximately 595.3 kb. The size of this mitogenome is typical among angiosperms, comparable to aquatic plants like *Nymphaea* (617.2 kb) (Dong et al., 2018) and *Nelumbo* (524.8 kb) (Gui et al., 2016). However, the presence of three independent circular chromosomes is uncommon in angiosperms. Previously, limitations in sequencing technology and assembly algorithms made it challenging to resolve plant mitogenome structures. However, advancements in PacBio HiFi and ONT R10 long-read sequencing technologies, coupled with the development of graphical assembly software like PMAT (Bi et al., 2024a), OATK (Zhou et al., 2025), TIPPo (Zhou et al., 2025), and HiMT (Tang et al., 2025), have enabled the resolution of an increasing number of plant mitochondrial structures. This progress lays a crucial foundation for deepening our understanding of the structural evolution of plant mitogenomes.

### Classification and characteristics of plant mitochondrial repetitive sequences

Generally, non-tandem repetitive sequences within plant mitogenomes serve as the driving force behind frequent recombination, constituting a key factor in their complex and diverse structures. The repetitive sequence characteristics of angiosperm mitogenomes allows categorization of repetitive sequences into three types based on length and recombination activity (Unseld et al., 1997; Wynn and Christensen, 2019): 1) Large-scale repeats (≥1000 bp), exhibiting the highest recombination activity and readily generating isomers or multiple subgenomes through recombination, a process typically occurring at high frequency and is reversible; 2) Medium-sized repeats (50–1000 bp), which generally exhibit low and irreversible recombination activity (Unseld et al., 1997). However, recombination events mediated by medium-sized repeats are often closely associated with environmental stimuli, plant growth and development, and phenotypic changes (Mackenzie and Kundariya, 2020); 3) Short-fragment repeats (<50 bp) exhibit the lowest recombination activity and primarily repair mutation sites via non-homologous end-joining mechanisms (Zou et al., 2022).

In this study, we conducted a comprehensive and in-depth analysis of repetitive sequences within the *C*. *demersum* mitogenome. Results revealed abundant non-tandem repeats (41,513) and tandem repeats (1,034), but relatively few simple sequence repeats. The prevalence of non-tandem repeats in *C*. *demersum* parallels that observed in two other aquatic angiosperms: *Nelumbo nucifera* (Gui et al., 2016) (2,376 repeats, including 33 >1 kb) and *Nymphaea colorata* (Dong et al., 2018) (nearly 200,000 repeats, but only 6 >1 kb). Compared to terrestrial angiosperms, aquatic angiosperms exhibit significantly increased non-tandem repeat sequences in their mitogenomes. Whether this correlates with their unique aquatic environment requires expanding the mitogenome dataset of aquatic angiosperms to accurately infer the evolutionary characteristics of repetitive sequences in these genomes. Additionally, we identified two dispersed repeats exceeding 1 kb in the *C*. *demersum* mitogenome, which mediate frequent recombination events on mtChr1 (contig26775: 22,042 bp) and mtChr2 (contig31447: 7,100 bp) (**Figure 1A**). However, for annotation convenience, only one possible conformation was exported in this study (**Figure 1B**). These two large repeat sequences may also recombine mtChr1 and mtChr2 into two smaller single-stranded chromosomes. To further elucidate the evolutionary drivers of plant mitogenomes, future studies should expand the number of mitogenomes in key plant groups using long-read sequencing (e.g., PacBio, ONT), analyze the repetitive sequence characteristics of complex mitogenomes, and investigate the fine-tuned role of repetitive sequences in regulating mitogenome stability through functional experiments (e.g., DSBR-related gene knockouts, recombination activity assays under stress conditions).

### PCG retention or loss in plant mitogenomes

**Figure 7:**
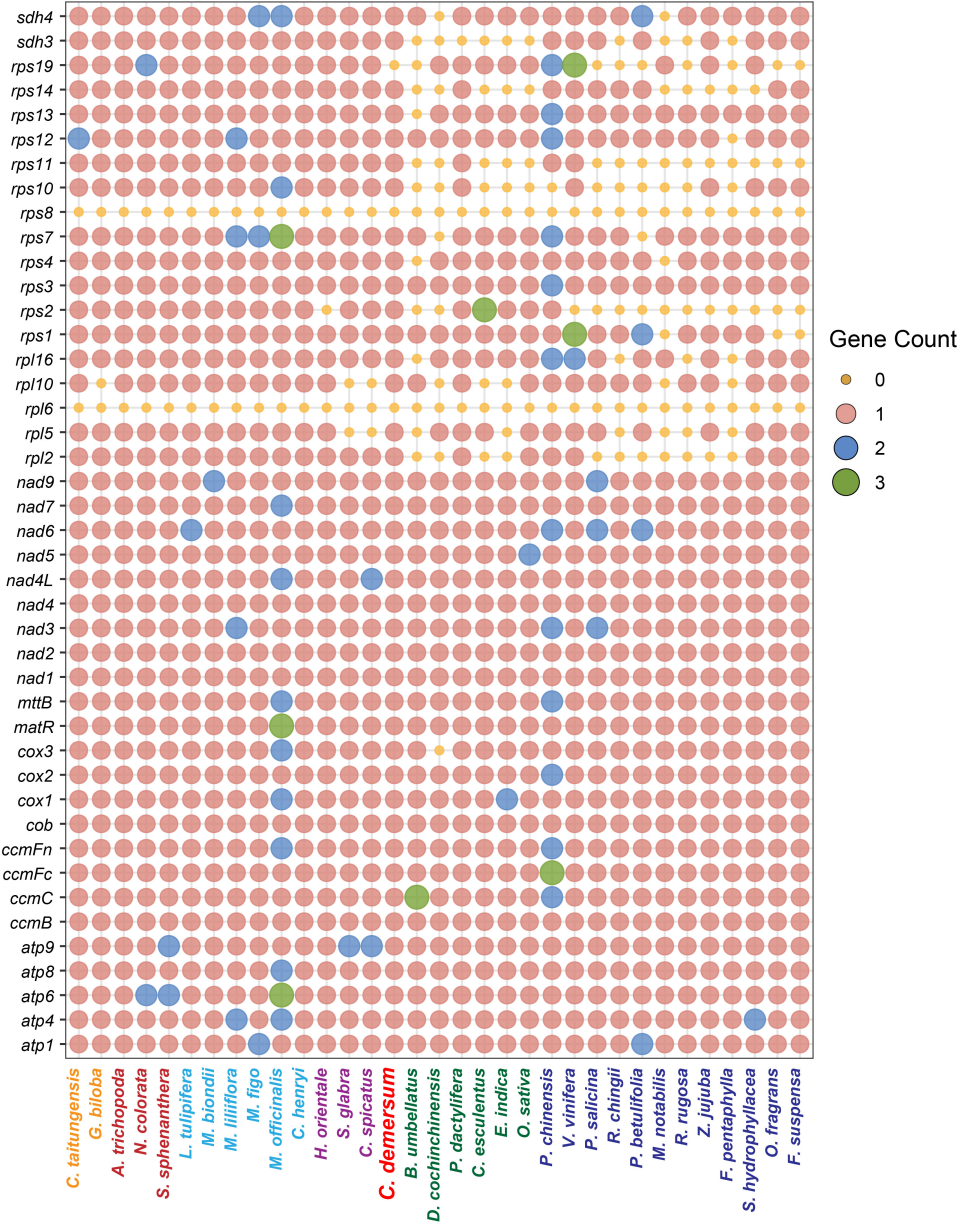
Protein-coding gene contents among 33 seed plant mitogenomes. The size and color of the circles represent the number of genes, while the color of the species names indicates different taxonomic groups.

During the progress of mitochondrial endosymbiosis, numerous mitochondrial PCGs have been lost or transferred to the nuclear genome. Even so, plant mitogenomes still preserve a specific set of PCGs that maintain the capacity for independent replication, transcription, and translation (Bi et al., 2024b). These retained PCGs, consisting of 24 core and 19 variable genes, primarily function to generate RNAs and proteins that are involved in oxidative phosphorylation and mitochondrial translation processes (Mower, 2020). Notably, among these PCGs, those encoding ribosomal proteins (SSU and LSU) and Complex II (*sdh3* and *sdh4*) subunits are more susceptible to being lost from plant mitogenomes. On the contrary, PCGs encoding other Complex subunits (Complex I, III, IV, and V) are much more likely to be conserved during the evolution (Adams et al., 2002). As shown in **Figure 7**, the ribosomal protein genes *rps8* and *rpl6* have been lost from all seed plant mitogenomes; however, this loss is not universal across all land plants (Mower, 2020): *rpl6* remains detectable in ferns (*Ophioglossum californicum* and *Psilotum nudum*), while both *rps8* and *rpl6* are still present in the lycophyte *Phlegmariurus squarrosus*. These observations indicate that *rps8* and *rpl6* were lost during the evolutionary transition from lycophytes and ferns to seed plants. In this study, all 24 core PCGs were identified in the *C. demersum* mitogenome, and only three variable PCGs (*rpl6*, *rps8*, and *rps19*) were lost during evolution (**Figure 7**). Similar to *C. demersum*, approximately 40 PCGs were identified in the mitogenomes of ANA, Magnoliids, and Chloranthales. Few PCG losses detected in these groups, with the exception of *rps8* and *rpl6*. In contrast, a substantial number of gene losses were observed in the large ribosomal subunit and small ribosomal subunit genes among monocots and dicots (**Figure 7**). These phenomena partially reflect the evolutionary position of *C. demersum* within angiosperms.

### Angiosperm phylogeny inferred from mitogenomes

The phylogenetic relationships among angiosperms are fundamental to understanding their origin and evolution; however, the relationships between the five major lineages of core angiosperms remain contentious (Yang et al., 2020b; Hu et al., 2023). Previous studies have predominantly relied on plastid or nuclear genomes (Li et al., 2019; Guo et al., 2022; Hu et al., 2023), while mitogenomes—despite their phylogenetic potential—have often been neglected owing to their structural complexity and low evolutionary rate. Recent studies suggest that the slow evolutionary rate of mitochondrial gene could help resolve deep angiosperm phylogeny. Xue et al. reconstructed a phylogeny using 38 mitochondrial genes from 108 taxa (Xue et al., 2021), while Lin et al. conducted phylogenetic analyses with 41 mitochondrial genes across 481 angiosperm species (Lin et al., 2025). Both studies consistently recovered a monophyletic clade comprising Ceratophyllales and Chloranthales, and resolved this clade as the sister group to eudicots—a phylogenetic pattern that is inconsistent with the APG IV system (The Angiosperm Phylogeny Group et al., 2016). In our study, the phylogenetic tree also supports the monophyly of Ceratophyllales and Chloranthales, but places this clade as sister to a combined monocot–eudicot clade, rather than solely to eudicots. These findings offer new insights and alternative hypotheses for reconstructing early angiosperm evolution. Advances in third-generation long-read sequencing and mitogenome assembly methods now enable more comprehensive phylogenomic studies using plant mitogenomes. Ultimately, the integration of nuclear, plastid, and mitochondrial data holds promise for resolving long-standing complex phylogenetic issues.

## Data Availability

The datasets presented in this study can be found in online repositories. The names of the repository/repositories and accession number(s) can be found in the article/**Supplementary Material**.
